# Elemental sulfur coarsening kinetics

**DOI:** 10.1186/s12932-014-0011-z

**Published:** 2014-08-06

**Authors:** Angel A Garcia, Gregory K Druschel

**Affiliations:** 1 Department of Geology, University of Vermont, Delehanty Hall, Burlington, 05405 VT USA; 2 School of Earth and Space Exploration, Arizona State University, Tempe, 85287 AZ USA; 3 Department of Earth Sciences, Indiana University-Purdue University Indianapolis, SL118, 723W. Michigan St., Indianapolis, 46202 IN USA

**Keywords:** Surfactant, Sodium Dodecyl Sulfate, Dynamic Light Scattering, Hydrogen Sulfide, Polysulfide

## Abstract

**Background:**

Elemental sulfur exists is a variety of forms in natural systems, from dissolved forms (noted as S_8(diss)_ or in water as S_8(aq)_) to bulk elemental sulfur (most stable as α-S_8_). Elemental sulfur can form via several biotic and abiotic processes, many beginning with small sulfur oxide or polysulfidic sulfur molecules that coarsen into S_8_ rings that then coalesce into larger forms:

1 $$S_{n}{O_{m}}^{2-}\to S_{8\;\left(\mathrm{aq}\right)}\to S_{8\;\left(\mathrm{nano}\right)}\to S_{8\left(\mathrm{sol}\right)}\to {S_{8}}_{\left(\alpha -S8\right)\;\left(\mathrm{bulk}\right).}$$

Formation of elemental sulfur can be possible via two primary techniques to create an emulsion of liquid sulfur in water called sulfur sols that approximate some mechanisms of possible elemental sulfur formation in natural systems. These techniques produce hydrophobic (S_8(Weimarn)_) and hydrophilic (S_8(polysulfide)_) sols that exist as nanoparticle and colloidal suspensions. These sols begin as small sulfur oxide or polysulfidic sulfur molecules, or dissolved S_8(aq)_ forms, but quickly become nanoparticulate and coarsen into micron sized particles via a combination of classical nucleation, aggregation processes, and/or Ostwald ripening.

**Results:**

We conducted a series of experiments to study the rate of elemental sulfur particle coarsening using dynamic light scattering (DLS) analysis under different physical and chemical conditions. Rates of nucleation and initial coarsening occur over seconds to minutes at rates too fast to measure by DLS, with subsequent coarsening of S_8(nano)_ and S_8(sol)_ being strongly temperature dependent, with rates up to 20 times faster at 75°C compared to 20°C. The addition of surfactants (utilizing ionic and nonionic surfactants as model compounds) results in a significant reduction of coarsening rates, in addition to known effects of these molecules on elemental sulfur solubility. DLS and cryo-SEM results suggest coarsening is largely a product of ripening processes rather than particle aggregation, especially at higher temperatures. Fitting of the coarsening rate data to established models for Ostwald ripening additionally support this as a primary mechanism of coarsening.

**Conclusions:**

Elemental sulfur sols coarsen rapidly at elevated temperatures and experience significant effects on both solubility and particle coarsening kinetics due to interaction with surfactants. Growth of elemental sulfur nanoparticles and sols is largely governed by Ostwald ripening processes.

**Electronic supplementary material:**

The online version of this article (doi:10.1186/s12932-014-0011-z) contains supplementary material, which is available to authorized users.

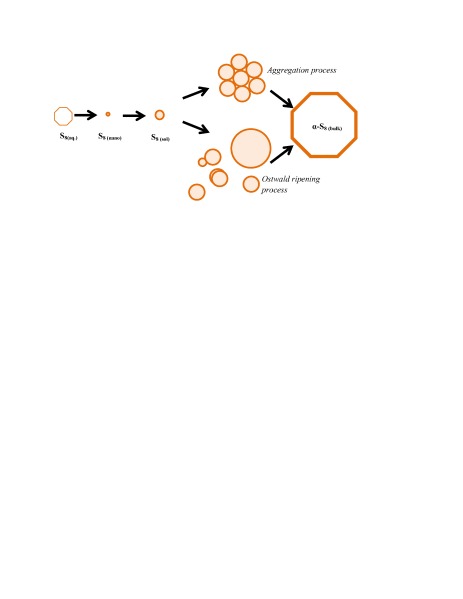

## Background

Sulfur is a key element associated with interactions between magma chambers, primary and meteoric water, country rock, and the atmosphere in hydrothermal systems, in additional to being important as an electron donor or acceptor for a range of microbial metabolisms [1]–[4]. Sulfur exists on Earth between the most reduced form, hydrogen sulfide (S^2−^) and the most oxidized form, sulfate (S^6+^). Sulfur polymerizes to form chains of sulfur molecules, and these chains can additionally cyclize to form rings [5],[6]. Sulfur reacts with different organic molecules to form a large number of different organic-S compounds [7], though for simplicity we will consider only inorganic S chemistry here. Many of these forms of sulfur interact strongly with different metal ions, especially important is the interaction between sulfide and metals to form a number of insoluble metal sulfide minerals [8],[9]. As electron transfers occur in one, or at most 2, electron steps, there are a number of intermediate sulfur forms involved with redox reactions between sulfide and sulfate [10]. Key dissolved intermediate sulfur compounds include polysulfides (S_x_^2−^, where x = 2-9 in most waters), thiosulfate (S_2_O_3_^2−^), sulfane monosulfonic acids (S_x_O_3_^2−^, where x can be from 3 to over 100 but mostly in the 3–12 range in most waters), polythionates (S_x_O_6_^2−^, where x = 3-6 in most waters), and sulfite (SO_3_^2−^). Several excellent reviews on detailed sulfur chemistry and geochemistry exist [3],[5],[8],[11],[12].

Elemental sulfur is the ground state, or atomic, form of sulfur, S^0^, and is most stable in aqueous systems as the mineral elemental sulfur in the α-S_8_ configuration (space group Fddd) [5],[13]. Sulfur in its atomic form (S^0^, here defined as a single S molecule with zero valence), should not be mistaken with mineral sulfur; S^0^ has a high enthalpy of formation and is not stable at ambient temperature [14],[15]. Sulfur atoms have a strong tendency to catenate, resulting in polymeric forms that can exist as rings or as chains of varying sizes and configurations, but is most stable as an 8-membered ring with a crown-shaped configuration (S_8_) [5],[13],[14],[16]. Elemental sulfur in solid form exists as about 30 different allotropes that have been characterized; each varying in viscosity, color, and melting point [5],[13]. These solid forms of elemental sulfur bring together these rings or chains with relatively weak van der Waals forces, this weak interaction makes elemental sulfur a soft mineral with a low melting point and high volatility [13]. α-S_8_ has a high refractive index (2.04) with high optical relief when transmitted light microscopy is used. The α-S_8(bulk)_ crystal has a large birefringence (0.29) that can be observed, even if they are only 0.5 μm thick [17],[18].

Elemental sulfur is a product of a number of reactions, including the oxidation of hydrogen sulfide [19] and the acid dissociation of thiosulfate and polysulfides [20]–[22]. This product does not start as the S^0^ atomic form, rather it is the result of a series of chain-lengthening reactions involving thiosulfate, sulfane monosulfonic acids, polythionates, polysulfides, and sulfite to generate at least a 9-sulfur chain that decomposes to S_8_[23]_,_ for example with thiosulfate via the reactions [23]:

2 $${\mathrm{HS}}_{2}{O_{3}}^{-}+\;S_{2}{O_{3}}^{2-}\to S_{3}{O_{3}}^{2-}+\;{{\mathrm{SO}}_{3}}^{2-}$$

3 $${\mathrm{HS}}_{8}{O_{3}}^{-}+\;S_{2}{O_{3}}^{2-}\to {\mathrm{HS}}_{9}{O_{3}}^{-}+\;{{\mathrm{SO}}_{3}}^{2-}$$

4 $${\mathrm{HS}}_{9}{O_{3}}^{-}\to \;S_{8\;\left(\mathrm{aq}.\right)}+{{\mathrm{HSO}}_{3}}^{-}$$

The dissolved 8-sulfur ring-form of elemental sulfur (S_8(aq)_), is sparingly soluble in water, but that solubility is strongly temperature dependent – S_8(aq)_ concentrations in equilibrium with mineral sulfur (α-S_8_) range from 6.1 nM at 4°C to 478 nM at 80°C [24]. Solubility of S_8(aq)_ is also affected strongly by the presence of surfactants; the solubility of sulfur was found to increase 5000-fold in the presence of several model surfactants by Steudel and Holdt [25]. This was interpreted to be related to the formation of micelles of surfactant-type molecules with an S_8_ ring in the hydrophobic interior of these micelles.

The solubility of elemental sulfur is probably most significantly impacted by the equilibrium between elemental sulfur and hydrogen sulfide or bisulfide to form polysulfide, via the reaction [23],[26]–[28]:

5 $$\left(n-1\right)/8\;\alpha -S_{8\;\left(\mathrm{bulk}\right)}+\;{\mathrm{HS}}^{-}\to \;{S_{n}}^{2-}+\;H^{+}$$

This reaction can constrain the solubility of elemental sulfur in many systems [29], where excess sulfide can quantitatively consume elemental sulfur [8]. The equilibrium constants for this reaction have been determined for temperatures up to 80°C [27],[30], showing equilibrium polysulfide activities to be predominant at more alkaline pH. The rate of forward reaction for this can be limited by surface area, as has been found to be the case for biologically produced sulfur [31]. The bioavailability of sulfur for microbial metabolisms may also be significantly affected by this reaction (4) with a role for S_8(nano)_ and polysulfides as a part of making different forms of sulfur more bioavailable [4],[32],[33].

S_8(aq)_ rings will quickly aggregate to form very small, but visible, forms of sulfur [11],[23],[34]. These forms are often in the tens to hundreds of nanometers to few micron size range of particles, do not settle from solution, and display particle scattering effects when suspended in solution [11]. These colloidal suspensions of elemental sulfur compounds have been termed ‘sols’ and consist of a range of particles of varying surface character and composition that can be formed via abiotic and biotic processes (for a review of this, see [11]). Hydrophobic (Weimarn sols, S_8(Weimarn)_) and hydrophilic (Raffo, S_8(Raffo)_; LaMer, and Selmi, collectively called Oden sols) sols have been described and have been reported to exist in an initial liquid state [11]. Additionally, sols have been observed to form from the acid decomposition of polysulfides (S_8(polysulfide)_), the reverse reaction of the solubilization of elemental sulfur with sulfide to form polysulfide (reaction 4) [23],[26],[29],[35]. The description of a liquid state for sols was based on physical macroscopic observations and the presence of a “Maltese-cross pattern” visible in cross-polarized reflected light optical microscopes at high magnification [11],[17], though this may not be definitive evidence of a true liquid state [17]. The question of crystallization kinetics for initially precipitated sulfur sols is important, but beyond the scope of this paper. It is noted that sols undergo coarsening and eventually form particles of observable size that are at least eventually crystalline [11], but exactly how and when that occurs is poorly constrained. Thus S_8(sol)_ and S_8(nano)_ may well be describing the same material under at least some conditions. Both hydrophobic and hydrophilic forms of sulfur sols are described to have a negative surface charge at circumneutral pH conditions, derived from anion sorption (hydrophobic sols) or the presence of significant long-chain S molecules (polysulfides to polythionates) as part of the sol itself (hydrophilic sols) [11],[15],[34]. Kleinjan et al. [15] measured the electrophoretic mobility of biologically produced elemental sulfur as 2.3, and Janssen et al. [36], noted a difference between the measured isoelectric point and point of zero charge due to an inhomogeneous charge distribution in the polymer layer.

As with other mineral systems in which single molecules or subunits coalesce towards bulk mineral sizes and atomic configurations; elemental sulfur must as well progress via a series of reactions towards the thermodynamically most stable form, α-S_8(bulk)_:

6 $$S_{n}{O_{m}}^{2-}\to \;S_{8\;\left(\mathrm{aq}.\right)}\to \;S_{8\;\left(\mathrm{nano}\right)}\to \;S_{8\;\left(\mathrm{sol}\right)}\to \;\alpha -S_{8\;\left(\mathrm{bulk}\right)}$$

Particle coarsening, the macroscopic observation of particles increasing in size, is a combination of processes that increase overall particle size and affect the distribution of particle sizes [37]. Transition from individual molecules to clusters to nanocrystals proceeds via classical nucleation theory (CNT) that requires the formation of a ‘critical nucleus’, a particle at a size where its rate of growth is greater than its rate of dissolution [38]. S_8(aq)_ rings are inherently hydrophobic and their interaction quickly forms clusters of S_8_ rings that become S_8(nano)_, and can additionally incorporate other hydrophobic molecules that may be present to form a “dirty” sulfur cluster [23]. In the sulfur system the rate of S_8(aq.)_ aggregation to form larger clusters and nuclei would be a key part of how the mineral coarsens. For the sulfur system there is no indication of what size the critical nucleus may be, though condensed phases of elemental sulfur have been measured at sizes as low as 30 nm [39]. Nucleation growth would then potentially become less important under mass conservation principles when the majority of material transitions from small dissolved S_8(aq.)_ and small clusters of S_8(aq.)_ units to S_8(nano)_ or S_8(sol)_. Coarsening would then be governed by some combination of Ostwald ripening or aggregation [40]. Ostwald ripening is a dissolution-precipitation mechanism where the growth of larger particles occurs at the expense of smaller particles due to differences in relative surface energetics [41]. Aggregation occurs via the attraction between separate clusters, critical nuclei, or larger particles to drive coarsening. Attractive forces include bonding, electrostatic interaction, dipole-charge, dipole-dipole, van der Waals interactions and hydrophobicity [38]. Given that sulfur sols and biologically produced elemental sulfur colloids are charged, aggregation must be a balance of attractive (van der Waals and hydrophobic) vs. repulsive (electrostatic) forces [15],[38]. Aggregation can also occur via oriented attachment, which allows for growth towards α-S_8_ to proceed without the need for recrystallization on the local scale [38]. Surfactants can affect the kinetics of elemental sulfur nanoparticle growth as well, a prior study on Raffo sols derived from thiosulfate acidification [42], indicated that surfactants play a significant role in the growth kinetics of elemental sulfur nanoparticles, with the cationic surfactant CTAB (cetyltrimethyammonium bromide) having the largest effect at 28°C. Chaudhuri and Paria also defined a critical micellar concentration, above which the coarsening rates do not continue to increase, suggesting a maximum coverage of surfactants that affects both the growth rate and the maximum particle size at 28°C [42].

This study looks to investigate the rates of coarsening of elemental sulfur formed as hydrophobic and hydrophilic sols, as a function of temperature, pH, and considering the effect of ionic and nonionic lipid molecules. In this study we will utilize the following definitions for different forms of sulfur: S^0^ as one molecule of atomic sulfur, S_8_ as a single ring of atomic sulfurs; S_8(diss)_ as a single ring dissolved in a non-polar solvent; S_8(aq)_ as the dissolved (in water) component of S_8_ rings; S_8(nano)_ as solid nanoparticles of sulfur; S_8(sol)_ as sols prepared according to Steudel and including S_8(Weimarn)_ as hydrophobic Weimarn sols derived from S_8_ dissolved in methanol mixed with water and S_8(polysulfide)_ as sols derived from acidification of polysulfides under anoxic conditions, and α-S_8_ as mineral elemental sulfur in its most stable state at 25°C and 1 atm pressure. We note that some of these forms may not be distinguishable under all conditions and times, and that there can be overlap (S_8(sol)_ for instance is often also S_8(nano)_ when the size is below 100 nm).

## Results and discussion

### Instrumental and experimental variability

Dynamic Light Scattering measurements are based on the hydrodynamic properties of nanoparticles in constant (Brownian) motion measured over a time interval, and are particularly suited to measurement of spherical nanoparticles and colloids less than 5 nm. Light scattered by these particles changes intensity in time based on this motion and that fluctuation can be fit to describe mean size, size distribution, volume distribution, and dispersity index [43]–[45]. Generally this does not work as well if the particles are changing size at a rate faster than the measurement window.

In order to first assess the general applicability of this method to investigate elemental particle coarsening kinetics we measured the replicability of standards and samples to determine both instrument error and experimental error that could be affected by a constantly changing particle population. A total of 111 replicate measurements of a 100 nm particle size standard control exhibited an arithmetic measurement mean of 99.35 nm with a standard error of the mean of 0.487 nm. The best-fit slope is 0.01365 ± 0.01522 with a coefficient of determination of 0.007319. The deviation from zero is not significant. Data include measurements with a maximum point of 119.8 nm, a median of 98.2 nm, and a minimum of 89.9 nm. The dispersity index for these same standards was measured to have a measurement mean of 0.0464 with a standard error of the mean of 0.0262. Experimental variability of a solution of changing particle size was described by performing five (5) replicates (A, B, C, D, & E) of Weimarn sol formation (*Experiment set #1*). Table 1 illustrates the variability in these measurements as replicates and for the dataset combined as a whole. We find that DLS measurements can resolve particle populations changing in time, and can reasonably resolve relatively small changes in mean size with minute resolution, but that a dynamic particle population cannot be resolved with the same precision as a static population of particles. We note that the instrument cannot resolve changes in time at the very beginning of the experiments, which corresponds to a condition where particle coarsening is occurring at a rate too fast for DLS measurements. The method of cumulants [43],[46] to interrogate the changing scattering signal cannot resolve changes that are very fast and the software returns this case as unresolvable – we do not report any data in which the particle population changed at a rate faster than the DLS is capable of resolving.

**Table 1:** **Experimental error associated with defining coarsening rates for 5 replicate experiments with the slope and 2-**σ **standard error for the fit for each experiment, and the slope and 2-**σ **standard error for the data from all 5 replicates used as on set of measurements to define the coarsening rate (in particle diameter as nanometers per minute)**

Set	Slope (nm/min)	2-σ standard deviation
A	1.66	0.0289
B	2.70	0.512
C	1.20	0.0860
D	−0.251	0.248
E	0.382	0.319
Total	1.063	0.205

### Coarsening rates

Two distinct domains of coarsening were observed, an initial rate where the rate of particle size change was too great to quantify using DLS (the first few minutes of each experiment), and an intermediate rate of relatively constant particle coarsening. A distinct bifurcation in the size v. time plots demarcates these regions, though the exact timing of this is only roughly constrained as the first interval the DLS is able to make a measurement. These domains are likely a switch from coarsening governed by classical nucleation theory and the development of a critical nucleus for each particle to coarsening governed by either Ostwald ripening or aggregation. However, given the rates the initial coarsening processes occur and the instrumental limitations described above, these measurements cannot determine the size at which this switch occurs.

### Coarsening as a function of temperature

**Figure 1:**
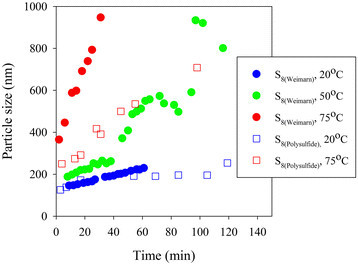
**Dynamic light scattering analysis of S**_**8(Weimarn)**_ **(solid symbols) and S**_**8(polysulfide)**_ **(open symbols) at different temperatures (20, 50, and 75°C).**

S_8(Weimarn)_ and S_8(polysulfide)_ coarsening rates were analyzed at different temperatures (*Experiment set #2*) (20, 50, and 75°C) (Figure 1). S_8(Weimarn)_ coarsening rate at room temperature (20°C) is 1.65 nm/min (±0.304 nm); at 50°C the coarsening rate is 6.62 ± 0.506 nm/min, and at 75°C the coarsening rate is 19.1 ± 0.875 nm/min. The difference between room temperature (20°C) and 50°C is 4.97 nm/min faster at 50°C. The rate of coarsening at 75°C is 11.6 times faster than room temperature (20°C). S_8(polysulfide)_ coarsening rate at room temperature (20°C) is 0.54 nm/min (±0.146 nm) for pH 3.1; at 75°C the coarsening rate is 5.51 ± 0.384 nm/min for pH 4.7. The differences in S_8(Weimarn)_ and S_8(polysulfide)_ coarsening rates likely reflect fundamental differences in the surface character of each particle, with the more hydrophobic S_8(Weimarn)_ particles exhibiting a substantially different temperature effect on rates at 75°C.

**Figure 2:**
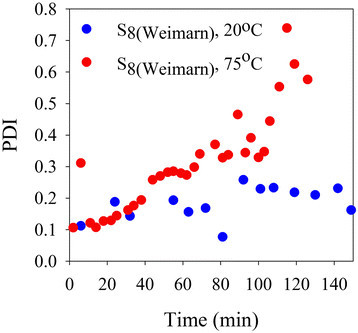
**Polydispersity index (PDI) analysis of S**_**8(Weimarn)**_ **at two different temperatures.**

Polydispersity index (PDI) was additionally measured to look at how the distribution of particle sizes change as a function of time and temperature (Figure 2). At 20°C the rate of change in dispersity is statistically not significantly different from zero indicating the particle size distribution stays uniform through time, consistent with Steudel’s [11] observation on Weimarn sols, an effect Steudel also observed to be sensitive to the specific preparation of the sol (water added to the methanol solution). Greater dispersity indices at higher temperatures suggest a mechanism of coarsening that may relate to specific conditions of Ostwald ripening [46],[47], though as a sole indicator this is insufficient to determine mechanisms.

The solubility of α-S_8_ increases with temperature [24],[48], including elemental sulfur nanoparticulates [6],[11],[12]. Weimarn [49] noted “disappearing” S_8(Weimarn)_ when temperature was increased. In our experiments where S_8(Weimarn)_ particles are synthesized at 75°C, the particles “disappeared” from solution by the time they reached approximately 3 μm in size after approximately 100 min. Solutions at 75°C become clear and the particle density in DLS instrument drops under the limit of detection. No precipitates were formed, even when the solution was cooled at room temperature (20°C), suggesting the rate of coarsening measured at elevated temperatures is a minimum rate that exceeds the rate of overall particle dissolution.

### Coarsening as a function of pH

**Figure 3:**
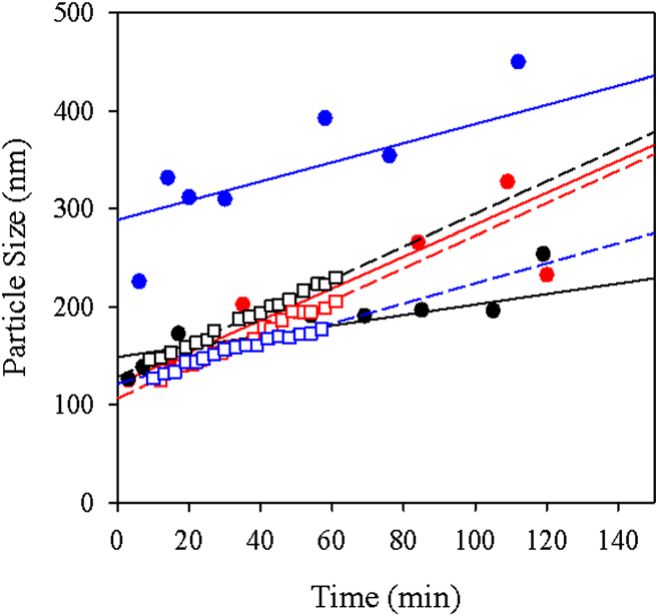
**Dynamic light scattering analysis of S**_**8(Weimarn)**_**(hollow symbols) at different pH (Black = 3,Blue = 7, and Red = 10), and S**_**8(polysulfide)**_**(solid symbols) at different pH (Black = 3, Blue = 7, Red = 9).** Best Fit lines included for S_8(Weimarn)_ (dashed lines) and S_8(polysulfide)_ (solid lines).

S_8(Weimarn)_ rate of coarsening was described at three different pHs (3, 7 and 10), and S_8(polysulfide)_ rate of coarsening was described at three different pHs (3,7,9) (*Experiment set #3*) (Figure 3). The rate of S_8(Weimarn)_ coarsening when solution is buffered to 3 is 1.666 ± 0.0288 nm/min At pH 7, the rate is 1.658 ± 0.0552 nm/min, while at pH 10 the rate of particle coarsening is 1.207 ± 0.05295 nm/min. pH measurements performed every 2 h confirmed there were no changes in pH over the course of the experiments. Statistical analysis shows that the difference between slopes is significant within the dataset but that the deviation is within that assessed previously for replicate error using DLS to describe particle systems changing in time. Since the pH zero point of charge (pH_zpc_) for elemental sulfur has been measured at 2.3 but with a heterogeneous charge due to surface polymerization [15],[36], these experiments were all under conditions where the particle was likely negatively charged and thus should experience electrostatic repulsion. The effect of pH on particle size coarsening for Weimarn sols thus suggests that pH does not significantly affect the rate of particle coarsening in Weimarn sols between pH 3 and 10. The rate at which S_8(aq.)_ coagulates is likely more dependent on the hydrophobicity of elemental sulfur particles, at least above the pH_zpc_. pH may have a more significant effect on the initial rates of coarsening for these particles, however, given that these rates are only constrained by 2 time points we will not attempt to quantify that effect with this dataset. pH does have a strong effect on rates of thiosulfate and polysulfide decomposition [20],[50]–[53], which in turn affects the rate of supply of S_8(aq)_ and subsequent coarsening.

### Coarsening as a function of surfactant presence

**Figure 4:**
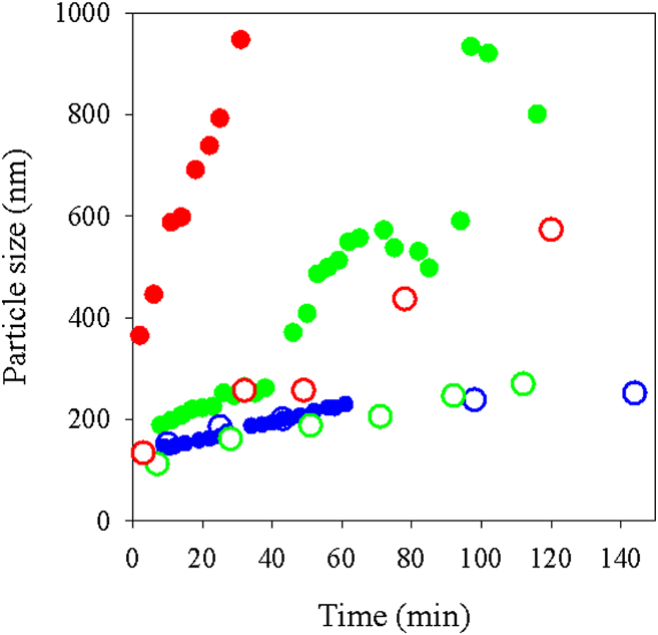
**Dynamic light scattering results illustrating effect of SDS surfactant on S**_**8(Weimarn)**_**particle coarsening kinetics at 3 temperatures.** Solid circles represent S_8(Weimarn)_ sols without SDS while hollow circles represent S_8(Weimarn)_ formed in the presence of SDS. Blue symbols are at 25°C, green symbols at 50°C, and red symbols at 75°C.

Sodium dodecyl sulfate (SDS) was used as a model ionic surfactant to investigate the effect of surfacants on sulfur nanoparticles over a range of temperatures, a compound previously used to describe the role of surfactants in solubilizing S_8(aq)_ from α-S_8(bulk)_[25] and the synthesis of S_8(nano)_ at room temperature [39] (Experiment set #4, Figure 4). The rate of S_8(Weimarn)_ coarsening on experiments performed at 20°C with SDS is 0.6911 ± 0.1268 nm/min while at 75°C the rate of coarsening is 3.533 ± 0.2212 nm/min. SDS slows the coarsening process more than two-fold at 20°C, but more than five-fold at 75°C. S_8(polysulfide)_ displays a rate of particle coarsening that also decreases when surfactant-type molecules are present; from 1.952 ± 0.1846 nm/min without SDS to 0.2169 ± 0.01016 nm/min with SDS at 20°C, a nine-fold decrease in the coarsening rate for S_8(polysulfide)._ These results are consistent with Chaudhuri and Paria [42] describing the effect of surfactants on S_8_(_Raffo_), and extend the role of surfactants to higher temperature regimes for sulfur sols applicable to hydrothermal systems.

**Figure 5:**
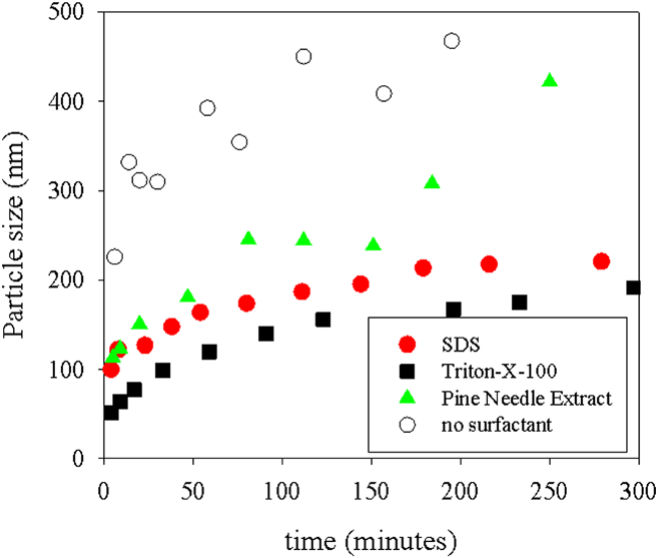
**Dynamic light scattering analysis of S**_**8(polysulfide)**_ **with different surfactant-type molecules (SDS, Triton-X-100), and Pine needle extract.**

To extend the thinking for these experiments towards application to terrestrial hydrothermal systems, we compared ionic (SDS) and non-ionic (Triton-X-100) surfactants to a complex organic extract derived from pine needles (Experiment set #5, Figure 5). Observations at several thermal springs in Yellowstone National Park (data not published) note the presence of significant amounts of pine needles as a possible external source of organic modifiers and surfactants. Because the pathways for elemental sulfur formation in these systems may be closer to one involving polysulfide, these three kinds of surfactant-type molecules (SDS, Triton-X-100, and pine needle extract) were used with S_8(polysulfide)_ sols (Experiment set #8; Figure 4, Table 2). Considering that the coarsening rate of S_8(polysulfide)_ in the absence of surfactants at these same pH, temperature, and solution constraints is 1.00 nm/min, pine needles can supply some component of micellar structure or other organic modifier to stabilize S_8(nano)_ and S_8(sol)_ in solution.

**Table 2:** **Summary of experimental conditions for all experimental sets**

Experiment set #	Surfactant	pH	Temperature (°C)	S_8 (sol)_
1	-	2.99	20	S_8 (Weimarn)_
1	-	2.98	20	S_8 (Weimarn)_
1	-	3.10	20	S_8 (Weimarn)_
1	-	2.99	20	S_8 (Weimarn)_
1	-	2.97	20	S_8 (Weimarn)_
2	-	3.09	20	S_8 (polysulfide)_
2	-	4.7	75	S_8 (polysulfide)_
2 & 4	-	2.98	20	S_8 (Weimarn)_
2 & 4	-	2.92	50	S_8 (Weimarn)_
2 & 4	-	2.63	75	S_8 (Weimarn)_
3	-	2.98	20	S_8 (Weimarn)_
3	-	6.99	20	S_8 (Weimarn)_
3	-	9.98	20	S_8 (Weimarn)_
3	-	3.09	20	S_8 (polysulfide)_
3	-	8.48	20	S_8 (polysulfide)_
3	-	6.7	20	S_8 (polysulfide)_
4	SDS	3.12	20	S_8 (Weimarn)_
4	SDS	3.05	50	S_8 (Weimarn)_
4	SDS	3.07	75	S_8 (Weimarn)_
5	SDS	5.7	20	S_8 (polysulfide)_
5	Triton-X-100	5.84	20	S_8 (polysulfide)_
5	Pine needle extract	5.6	20	S_8 (polysulfide)_
6	-	2.96	20	S_8 (Weimarn)_
6	-	3.09	20	S_8 (polysulfide)_

### Cryo scanning electron microscopy

**Figure 6:**
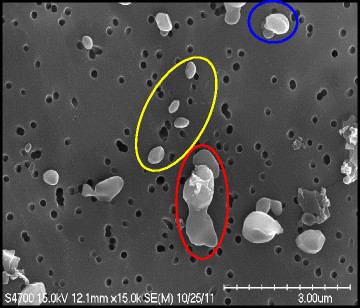
**SEM image from S**_**8(Weimarn)**_**particles.** Suggested Ostwald ripening mechanisms: (Blue) Collision interaction, (Red) Collapse interaction, and (Yellow) particles that are not interacting.

Cryogenic scanning electron microscopy images from S_8(Weimarn)_ experiments (Figure 6) suggest that the coarsening process occurring with this kind of sulfur sols is an Ostwald ripening process. The Ostwald ripening process is described as the exchange of volume by the mechanisms of collision and particle collapse [54]. Images from synthesized particles (Figure 6) illustrate collisions (blue circle), non-interaction (yellow circle), and the collapse (red circle) of particles.

### Models of particle coarsening

**Figure 7:**
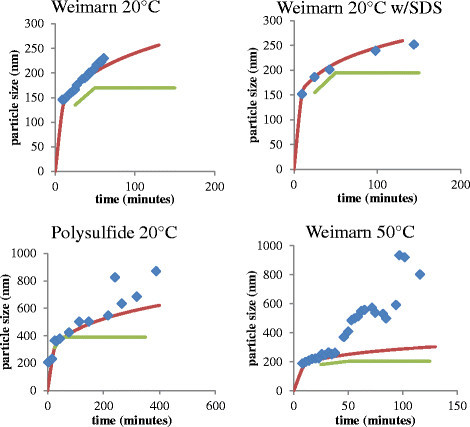
**Example fits for selected experimental data with Ostwald ripening models utilizing rate constants k and exponential fits from Table**3**.** Blue diamonds represent data experimental data, red lines represent the fits for the Ostwald ripening model (equation 6), and green lines represent the fits for the aggregation model (equation 7), using experimentally derived parameters k and n (Table 3).

Coarsening of sulfur sols likely occurs as either Ostwald ripening or aggregation after an initial stage during which the formation of critical nuclei would form. Analysis by DLS misses the initial stages of this coarsening because the rate of these initial steps are much faster than DLS techniques are able to capture at these conditions. However, we can investigate the quantifiable portion of these processes by fitting our coarsening rate data to standard models describing Ostwald ripening and aggregation based growth [41],[55],[56], as has been done with ZnS and FeS nanoparticles [56],[57]. We utilized an Ostwald ripening model:

7 $$D\left(t\right)\;=\;D_{0}+\;k*t^{1/n}$$

where the particle size at time t, D(t), is a function of the initial particle size (D_0_, in this case D_0_ equals zero) and a rate constant k with an exponent n that can be calculated for each experimental condition by plotting the natural log of the time and the natural log of the particle size [41],[56],[57]. Results of this analysis to derive k and n for experimental sets including S_8(Weimarn)_ and S_8(polysulfide)_ sols are listed in Table 3. The calculated value of n suggests physical meaning in terms of the coarsening rate; the rate of the growth may be controlled by diffusion in solution (n ≈ 1), diffusion at the particle surface (n ≈ 2), or the interface dissolution/precipitation step (n ≈ 3) [41]. Higher order exponents can indicate crystal growth controlled by diffusion on the grain/matrix boundary (n ≈ 4) or crystal growth controlled by dislocation-pipe diffusion (n ≈ 5) [57]. Results for this Ostwald ripening exponent for Weimarn sols at different temperatures, with and without SDS as a surfactant molecule, and for polysulfides (Table 3) suggest coarsening may be controlled at the particle boundary, with higher temperature generally resulting in more diffusional control. Figure 7 shows 4 examples of sol coarsening experiments with the Ostwald ripening model data; fits for these do show deviations from the model, and the fits get worse with higher temperature but are not significantly impacted by the presence of SDS as a model surfactant. A model describing aggregation-based coarsening was also developed [41],[56]:

8 $$D\left(t\right)=\frac{D_{0}\left(\sqrt[{3}]{2}\mathrm{kt}+1\right)}{\left(\mathrm{kt}+1\right)}$$

**Table 3:** **Linear regression results of ln time v. ln particle size plots of selected experiments for fitting to standard models describing Ostwald ripening and aggregation based growth of sulfur sols**

Sol type	T (and surfactant)	k	n
Weimarn	20	77 ± 4.0	3.9 ± 0.21
Weimarn	50	116 ± 6.4	4.5 ± 0.32
Weimarn	75	266 ± 24	3.0 ± 0.26
Weimarn	20 & SDS	99 ± 2.7	5.3 ± 0.18
Weimarn	50 & SDS	60 ± 6.5	3.3 ± 0.26
Weimarn	75 & SDS	73 ± 24	2.5 ± 0.36
Polysulfide	20	139 ± 20	3.6 ± 0.34

and fits for this model also calculated and plotted in Figure 7. These aggregation model fits do not fit the data as well, suggesting Ostwald ripening may be more of a controlling process for sol coarsening in these experiments. Deviation of these models from experimental data is likely due to multiple processes occurring over the time series under investigation. Notable is that the fits are worse for progressively higher temperatures; the dissolution of elemental sulfur during the course of the experiments is an additional variable that these models do not account for.

### Experimental

Experiments probing the coarsening kinetics of sulfur sols at different conditions are summarized in Table 2. Elemental sulfur as S_8(aq)_, S_8(Weimarn)_, S_8(polysulfide)_, were prepared from Precipitated Sulfur (from Fisher scientific, Lot. No. 028783) according to established techniques [11],[25]. X-ray diffraction of the precipitated sulfur confirmed pure α-S_8_ as the starting material. S_8(diss)_ is produced by dissolving elemental sulfur powder α-S_8(bulk)_ in a strong organic solvent [49]. S_8(diss)_ was prepared for these experiments by adding 15 g (excess) of precipitated sulfur in 100 ml methanol and stirring it for several days, then decanting the supernatant and filtering through a hydrophobic 0.2 micron filter, producing a clear solution. The resulting S_8(diss.)_ solution was analyzed with DLS to ensure that the solution does not have any measureable particles. Hydrophobic sols (S_8(Weimarn)_) was prepared by pouring 15 ml saturated α-S_8(bulk)_ methanol solution into 500 ml of 18 MΩ water [11],[49]. S_8(polysulfide)_ sample solutions were prepared daily for each experiment by acidification of a polysulfide solution with HCl. Sodium pentasulfide salts were synthesized using methods adapted from Rosen and Tegman [58]. Briefly, polysulfide salts were prepared by reacting 0.95 g anhydrous sodium sulfide with 1.55 g crystalline elemental sulfur that had been dried in an oven at 80°C. All preparation and handling of the polysulfide salts were done in a dry anoxic glove box. Reagents were mixed together by grinding, placed in quartz tubes, and sealed under an atmosphere of N_2_ before evacuation on a vacuum line and sealing of the quartz glass using an acetylene torch. Synthesis took place through melting and reaction for 12 h at 210°C, followed by an annealing step for about half an hour at 350°C, removal and regrinding of the product under an N_2_ atmosphere, replacement of the mixture into another glass tube, and a final melting and reaction step at 210°C for 10 h. The salts were then washed with hexane to remove residual elemental sulfur impurities, resealed under vacuum, and kept at −20°C in the dark until needed.

Surfactants were used to emulate environments where organic material is present at the moment of S_8(nano)_ formation. Surfactants were dissolved in water before performing experiments. Sodium dodecyl sulfate (SDS, an ionic surfactant) and Triton-X-100 (a non-ionic surfactant) were used in these experiments. Additionally, pine needles were collected from an area surrounding Cinder pool, (in Yellowstone National Park) and used as a source of organic material. Pine needles were soaked in 18 MΩ water acidified with HCl to pH 4 at 50°C overnight. After cooling the solution was filtered using 0.2 μm filter paper and analyzed with DLS, no particles were detected.

For each S_8(sol)_ preparation pH was buffered and monitored throughout the experiment (measured used a Fisher accumet pH meter and combination electrode). For S_8(Weimarn)_ experiments, 5 mM of buffering salts were added to avoid any significant changes in pH. Potassium hydrogen phthalate (C_8_H_5_KO_4_, pKa = 2.95), PIPES (C_8_H_18_N_2_O_6_S_2_, pKa = 6.76) and sodium bicarbonate (NaHCO_3_, pKa = 10.24) were used to adjust the pH in the aqueous solution. After the addition of salts 3 M HCl or 1 M NaOH was used to set the pH. Potassium hydrogen phthalate was not used with SDS because K^+^ from the salt makes an insoluble precipitate of potassium SDS. Sulfur sols formed by the decomposition of polysulfides (S_8(polysulfide)_) were not buffered; pH for these experiments was adjusted to 8.48, 3.09, 6.7, and 4.7 without the addition of buffers. S_8(polysulfide)_ experiments with surfactant were adjusted to pH 5.6, 5.7, and 5.84. pH was measured every 10 minutes using a calibrated pH electrode.

Temperature for all the experiments was controlled by the use of a water bath. Buffered pH solutions (water with salts) were thermally equilibrated for at least 30 min in a water bath at desired temperature before performing any experiment, including room temperature experiments. Temperature was constant throughout DLS measurements; the DLS instrument compartment is thermostated and was set at desired temperature to avoid any fluctuations in temperature that could affect the rate of coarsening of S_8(sol)_.

## Conclusions

The kinetics of S_8(Weimarn)_ and S_8(polysulfide)_ particle coarsening is strongly temperature dependent, and can be significantly impacted by the presence of surfactants and other organic modifiers. We extend the temperature range of defined sulfur particle coarsening to 75°C, and show that for higher temperature conditions found in many terrestrial and marine hydrothermal systems, the coarsening rate of sulfur is very rapid. Consistent with other studies on Raffo sols [39],[42], this investigation of Weimarn sols and sols produced from acid decomposition of polysulfides are affected by ionic and nonionic surfactants in aqueous solutions. The presence of surfactant-type molecules in solution not only affects the solubility of S_8(aq)_ in equilibrium with α-S_8(bulk)_[25], but also affects the size of sulfur nanoparticles. These parameters potentially affecting the size of elemental sulfur in hydrothermal systems may also have significant impact on other reactions involving elemental sulfur, especially the reaction between elemental sulfur and hydrogen sulfide to form polysulfides, and subsequent reactions that can affect intermediate sulfur species bioavailability [4].

Coarsening of S_8(nano)_ and S_8(sol)_ proceeds via a combination of classical nucleation, Ostwald ripening, and aggregation. Coarsening rate data fit to models describing both Ostwald ripening and aggregation processes [41],[56],[57] suggest Ostwald ripening is a key process governing elemental sulfur coarsening. Deviation from these fits may be due to multiple processes (i.e. a combination of aggregation and Ostwald ripening) and/or a combination of particle aggregation with dissolution that is particularly important at higher temperature where the model fits departed more significantly. Polydispersity index measurements of the particle size distribution at higher temperature indicated a more heterogeneous size distribution that may also suggest Ostwald ripening as a key process. Additionally, particle shape and evidence for particle interaction using Cryo-SEM results suggest that the main coarsening process is also *via* Ostwald ripening mechanisms (collision and collapse) [54].

## Methods

Dynamic light scattering (DLS) was employed to monitor particle size kinetics using a Beckman-Coulter DelsaNano C, which utilizes photo correlation spectroscopy (PCS), where size is estimated by averaging the rate of fluctuations in laser intensity scattered by particles that are diffusing in a liquid [43]. DLS can determine mean size, size distribution, volume distribution, and molecular weight, and polydispersity index (PDI, a measurement of the distribution of particle sizes in a sample) [43],[44],[59]. The method of cumulants [45],[59] is one of several ways that we can use the DLS data to improve particle characterization based on a statistical accumulation generation function about the mean particle size, intensity, and time. The Delsa Nano software utilizes the CONTIG algorithm to determine particle size distributions, based on an inverse Laplace transformation. Every DLS measurement for these experiments was set to capture 60 accumulations in a cuvette holder within the thermostated sampling compartment of the DelsaNano C.

Scanning electron microscopy (SEM) was used to image a sample of the Weimarn sols; because elemental sulfur is volatile, cryogenic conditions are the only reliable way to investigate the morphology of these particles. A Hitachi S4700 Field Emission Scanning Electron Microscope (FE-SEM) with a Gatan Alto 2500 Cryotransfer System and an Oxford INCA Energy (EDS) System was utilized at the University of Delaware Biotechnology Institute. Samples were filtered using a 0.2 μm filter using a vacuum unit filter (glass). Once the sample was collected, it was put on a SEM stage and frozen using liquid nitrogen. Once the stage and sample were completely frozen, they were inserted in the SEM and analyzed.

Linear regression models followed by one-way ANOVA tests were performed using GraphPad Prism software, version 6.00 for Mac OS X and SigmaPlot 11.0 for PC.

## Authors’ original submitted files for images

Below are the links to the authors’ original submitted files for images. Authors’ original file for figure 1 Authors’ original file for figure 2 Authors’ original file for figure 3 Authors’ original file for figure 4 Authors’ original file for figure 5 Authors’ original file for figure 6 Authors’ original file for figure 7
