# Supplementary figures and images for: Elemental sulfur coarsening kinetics
Source: Geochem Trans. 2014 Aug 6;15:11. doi: 10.1186/s12932-014-0011-z (PMC4631715; doi:10.1186/s12932-014-0011-z)

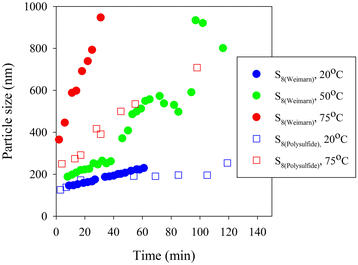

Supplement: Supplementary file 1 — Authors’ original file for figure 1 [file 12932_2014_11_MOESM1_ESM.gif]

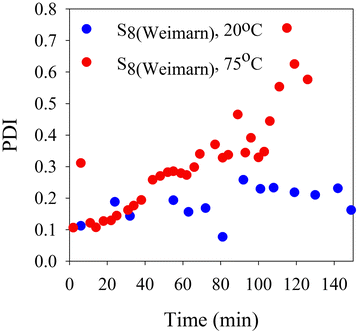

Supplement: Supplementary file 2 — Authors’ original file for figure 2 [file 12932_2014_11_MOESM2_ESM.gif]

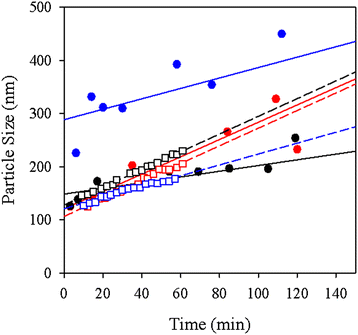

Supplement: Supplementary file 3 — Authors’ original file for figure 3 [file 12932_2014_11_MOESM3_ESM.gif]

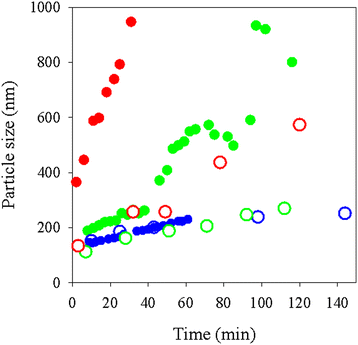

Supplement: Supplementary file 4 — Authors’ original file for figure 4 [file 12932_2014_11_MOESM4_ESM.gif]

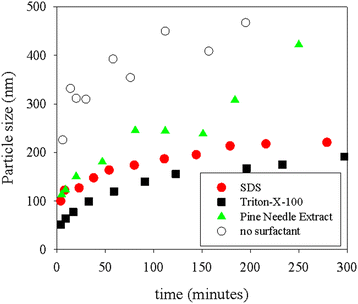

Supplement: Supplementary file 5 — Authors’ original file for figure 5 [file 12932_2014_11_MOESM5_ESM.gif]

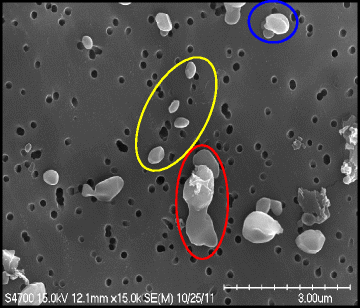

Supplement: Supplementary file 6 — Authors’ original file for figure 6 [file 12932_2014_11_MOESM6_ESM.gif]

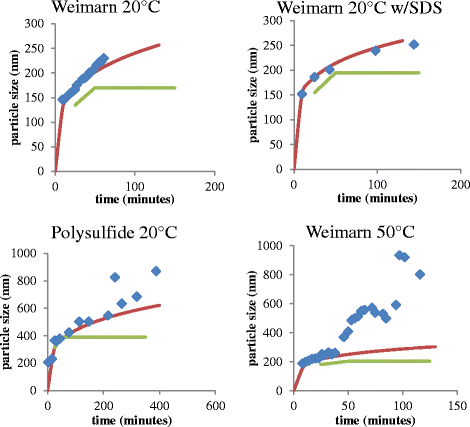

Supplement: Supplementary file 7 — Authors’ original file for figure 7 [file 12932_2014_11_MOESM7_ESM.gif]
